# Chairside Diabetes Screening: A Survey of Dental Providers at the Largest Municipal Healthcare System in the United States

**DOI:** 10.3290/j.ohpd.b2448635

**Published:** 2021-12-18

**Authors:** Nadia Laniado, Megan A. Cloidt, Victor M. Badner

**Affiliations:** a Dentist, Albert Einstein College of Medicine, Health+Hospitals/Jacobi Medical Center, Department of Dentistry/OMFS, Bronx, NY, USA. Conception and design, acquisition of data, or analysis and interpretation of data; drafted the article or revised it critically for important intellectual content; agreed to submit to the current journal; gave final approval of the version to be published; agreed to be accountable for all aspects of the work.

**Keywords:** diabetes, glucose monitoring, health promotion, interprofessional relations, primary health care

## Abstract

**Purpose:**

To assess the knowledge and attitudes of dentists at the largest municipal healthcare system in the United States with regard to point-of-care chairside diabetes testing in the dental clinic.

**Materials and Methods:**

A 29-item survey was designed in a web-based platform (Survey Monkey) and distributed in November 2020 to 264 dental providers (attending dentists and residents) within eleven dental departments of the New York City Health + Hospitals municipal healthcare system. The questionnaire included sections on demographics, current practices, level of diabetes training, scope of practice, and attitudes regarding chairside diabetes testing. Descriptive statistics, bivariate analyses, and logistic regression analyses were performed, with statistical significance set at 0.05.

**Results:**

Dentists’ willingness to provide chairside HbA1c screening was positively associated with their agreement that this was part of their role (OR = 7.2, p = 0.001) and that screening has an impact on diabetes control (OR = 4.3, p = 0.04). The two most commonly reported barriers to willingness to provide chairside HbA1c screening were amount of time required to obtain and discuss a patient’s test results (82.3%) and lack of reimbursement (70.6%).

**Conclusion:**

Among the largest municipal healthcare systems in the US, there is very limited integration of diabetes screening and management in the dental setting. Given the epidemic of diabetes among the vulnerable population that these hospitals serve, the dental setting is a critical but underutilized site for diabetes screening and medical referral. Increased efforts should be directed towards implementing workflows that incorporate auxiliary dental staff in order to address barriers to chairside screening.

Diabetes is a chronic disease with no or very mild symptoms in its early stages.^10,33^ By the time it is diagnosed, substantial damage to the cells that store and release insulin has often already occurred.^5^ For individuals with prediabetes, if their disease is left untreated, 15%-30% progress to type 2 diabetes within five years.^42^ It is critical therefore to understand not only the factors that may influence the progression of diabetes, but the early signs of disease. Evidence suggests that periodontal changes are the first clinical manifestation of diabetes and that interventions which improve the oral health of individuals with diabetes may lead to a decrease in diabetes severity, as measured by a lowering of glycated hemoglobin A1c (HbA1c) levels.^11,13^

In the United States (US), according to the National Diabetes Statistical Report 2020, over 34 million people have diabetes mellitus and another 88 million people have prediabetes.^34^ In addition, because it is asymptomatic in early stages, nearly 25% of adults with diabetes are undiagnosed.^28^ Both diabetes and periodontal disease are chronic diseases marked by systemic increases in mediators of inflammation, such as Interleukin 6 (IL-6) and C-reactive protein.^27^ There is a well-established bidirectional association between periodontal disease and diabetes mellitus: individuals with diabetes are at greater risk of periodontal disease, and similarly, individuals with chronic periodontal inflammation are at greater risk of altered glycemic control.^14,19,37,43^

Given the epidemic of diabetes and the high prevalence of undiagnosed disease, early detection is critical in order to improve health outcomes. Point-of-care testing by a dental provider can be performed by drawing capillary blood from a finger prick and patient fasting is not required. Administration of point-of-care HbA1c testing is within the scope of practice by dentists in New York State, yet implementation has been negligible. This is unfortunate, because for many vulnerable individuals, their only point of contact with the health care system may be their dentist,^40^ and this fast and simple screening test has enormous potential to identify those with undiagnosed prediabetes and diabetes.^45^

A recent study estimated that among the 10.2 million people who had a routine dental visit last year in the US, 1.6 million were not aware they were at risk for prediabetes or diabetes.^1^ This is a public health concern in the US, given that obesity is a risk factor for type 2 diabetes and there is an ongoing obesity epidemic.^36,41^ Many adults in the US do not have a yearly medical visit, but may have routine dental appointments, and thus dentists can play a critical and often overlooked role in primary preventive measures for diabetes.^4,44^ Therefore, understanding the willingness of dental providers to provide chairside screening to check for dysglycemia, especially at safety-net municipal hospitals, is critical prior to designing and implementing acceptable chairside diabetes screening protocols.

The aim of this study was to examine the knowledge and attitudes of dental professionals regarding chairside HbA1c screening at the largest municipal healthcare system in the US, serving a large segment of ethnically diverse and vulnerable populations with prediabetes and diabetes. We hypothesized that lack of knowledge regarding point-of-care testing and perceived roles were significant barriers in providers’ willingness to screen for diabetes.

## Materials and Methods

### Study Design

This study involved the administration of a survey instrument to attending and resident dentists in the dental departments of the eleven New York City municipal hospitals to examine their knowledge, attitudes, willingness and perceived barriers surrounding point-of-care diabetes screening in the dental clinic. Faculty dentists and dental residents from the New York City/Health + Hospital dental departments of Jacobi, North Central Bronx, Lincoln, Harlem, Metropolitan, Coney Island, Kings County, Queens, Elmhurst, Woodhull, and Bellevue Hospitals were recruited by e-mail for participation in the survey tool. Collectively, these inner-city hospitals provide the safety net for New York City residents, the vast majority of whom are individuals of color and low socioeconomic status. As providers in public health settings, the survey participants represented providers who were potentially the most critical for and amenable to population screening for prediabetes and diabetes.

Participants’ e-mail addresses were obtained from the chairperson of each hospital’s dental service. The e-mail described the purpose of the study, the amount of time needed to complete the survey, and assurances of participant anonymity. The survey examined the perceived barriers as well as the knowledge, attitudes, beliefs and willingness of dental providers to offer chairside HbA1c rapid testing in the dental care setting. Each respondent received a $15 amazon gift card as an incentive for completing the survey.

### Survey Instrument

A 29-item survey instrument was developed by adapting questions from the validated survey by Anders et al^3^ for examining attitudes of dental students towards diabetes screening. The survey included specific items, informed by the Theory of Planned Behavior, to assess and understand dentists’ attitudes, as well as impeding and facilitating factors, related to HbA1c screening.^17^ Survey questions were formulated to prompt either dichotomous or Likert-scale responses (ranging from 1 = “strongly agree” to 5 = “strongly disagree”). Five questions assessed respondents’ individual sociodemographic information including age, sex, current position (attending or resident), dental specialty (general practice, pediatric dentistry, oral surgery, orthodontics, endodontics, periodontics, dental public health, and dental anesthesia), and year of graduation from dental school. Six questions assessed respondents’ level of diabetes training and current provision of chairside diabetes monitoring.

The survey included 18 questions regarding scope and responsibility, barriers, and glucometer use. The 10-item scope and responsibility scale comprised the following items/questions. “Is it the dental professional’s responsibility to…”: (1) educate patients about the risks of diabetes to overall health and well-being?; (2) educate patients about the risk of diabetes to oral health?; and “Is it within the scope of dental practice to…”: (3) ask patients if they have diabetes? (4) advise diabetic patients to monitor their own blood glucose using a glucometer? (5) discuss benefits of controlling diabetes? (6) discuss specific strategies for controlling diabetes? (7) refer a patient for medical evaluation if the patient’s blood glucose is too high? (8) identify patients who may benefit from interventions to prevent/control diabetes? (9) take a diabetic patient’s blood glucose reading using a glucometer? and (10) screen for diabetes using a glucometer on patients who are not diagnosed with diabetes?

The 6-item barrier scale comprised the following items, starting with the assertion “The following are potential barriers to evaluation and counseling regarding elevated blood glucose”: (1) time, (2) lack of reimbursement for time to discuss patient’s blood glucose levels, (3) lack of confidence, (4) patient resistance, (5) lack of referral knowledge, and (6) lack of insurance reimbursement. Two survey items with a dichotomous response (yes or no) assessed point-of-care screening: (1) monitoring HbA1c levels in the dental office can impact controlling diabetes, and (2) I would be willing to participate in a training program to promote and administer chairside diabetes screening in my hospital’s dental department.

### Data Collection and Analysis

Data were collected in the secure SurveyMonkey platform and stored on a password-protected computer. Respondents’ e-mail addresses were collected in order to send the electronic gift card. Once the cards had been distributed, anonymized survey data were stored in an Excel file. No subject identifiers were stored. Completion of the survey implied consent. A four-week timeframe was allotted for return of the completed survey. Reminders were sent to the recipients at the end of weeks one, two, and three to encourage completion of the survey. The survey was pilot tested among a convenience sample of practitioners for length, readability, clarity and consistency.

The outcome of interest was willingness to screen for diabetes. This was measured by a survey item asking how willing providers would be to offer chairside HbA1c testing in the next year, based on 5-point Likert scale responses dichotomized to “strongly agree/agree” and “strongly disagree/disagree.” The main exposure of interest was a categorical variable that queried dental providers’ agreement that a dentist’s role should include diabetes screening. Additional covariates included individual demographic variables, number of hours of diabetes training, use of medical history forms that ask about diabetes, years in practice, dental specialty, and familiarity with the Code on Dental Procedures and Nomenclature (CDT) for point-of-care testing.

Data analysis included descriptive statistics, bivariate analyses, and multivariate logistic regression. Univariate analyses provided summary statistics of respondent characteristics, measures of dental providers’ willingness to perform finger-prick tests, dental professional’s agreement that screening for diabetes should be part of their role and associated independent variables. Crude associations between each independent variable and the dependent variable were examined by bivariate analysis using chi-squared and Fisher’s exact tests. The multivariable logistic regression model included the independent variable of interest as well as covariates demonstrating a bivariate association with the dependent variable at a level of p < 0.25, following the method of Hosmer and Lemeshow.^26^ Selected sociodemographic variables (age and sex) were included in the model regardless of p-values. All data analyses were conducted using statistical software Stata 13.0 (Stata; College Station, Texas). The significance level was set at p < 0.05. The study was reviewed by the Albert Einstein College of Medicine Institutional Review Board and was deemed exempt.

## Results

Table 1 presents the overall descriptive statistics, as well as participant characteristics by willingness to provide HbA1c screening in the dental care setting. The response rate to the survey was 51.5% (136/264). Overall, the study sample was predominantly male (61.0%) and between the ages of 25 and 34 (67.7%). Almost half, 48.5%, were general practice dentists and 34.6% were oral surgeons. The majority (86.0%) reported that their patient’s health history included questions about diabetes. With regard to updating diabetes status in the patient’s chart, 58.8% reported that they always or usually update, 27.9% sometimes update, and 13.2% never update diabetes status. Only 22.1% of respondents reported that they always or usually communicate with primary care providers regarding diabetes management of their patients.

**Table 1:** Characteristics of study participants overall and by willingness to provide HbA1c screening in the dental care setting, 2021, N = 136

Characteristic	Overall	Willing	Not willing	p-value**
		N (column %)		
Total N	136*	112 (82.4)	24 (17.7)	
**Age (years)**				0.60
18–24	1 (0.7)	1 (0.9)	0	
25–34	92 (67.7)	74 (66.1)	18 (75.0)	
35–44	13 (9.6)	11 (9.8)	2 (8.3)	
45–54	7 (5.2)	5 (4.5)	2 (8.3)	
55–64	16 (11.8)	15 (13.4)	1 (4.2)	
65+	6 (4.4)	5 (4.5)	1 (4.2)	
**Sex**				0.57
Female	51 (37.5)	44 (39.3)	7 (29.2)	
Male	83 (61.0)	66 (58.9)	17 (70.8)	
**Dentist’s position**				0.75
Resident	90 (66.2)	75 (67.0)	15 (62.5)	
Attending	44 (32.4)	35 (31.3)	9 (37.5)	
**Dentist’s specialty**				0.47
General practice	66 (48.5)	55 (49.1)	11 (45.8)	
Endodontics	1 (0.7)	1 (0.9)	0	
Periodontics	3 (2.2)	3 (2.7)	0	
Oral surgery	47 (34.6)	37 (33.0)	10 (41.7)	
Dental public health	3 (2.2)	3 (2.7)	0	
Prosthodontics	1 (0.7)	0	1 (4.2)	
Dental anesthesia	12 (8.8)	11 (9.8)	1 (4.2)	
Pediatric dentistry	3 (2.2)	2 (1.8)	1 (4.2)	
**Health history includes questions about diabetes**				0.90
No	9 (6.6)	8 (7.1)	1 (4.2)	
Yes	117 (86.0)	95 (84.8)	22 (91.7)	
Not sure	9 (6.6)	8 (7.1)	1 (4.2)	
**Diabetes status updated in chart**				0.48
Always	29 (21.3)	26 (23.2)	3 (12.5)	
Usually	51 (37.5)	39 (34.8)	12 (50.0)	
Sometimes	38 (27.9)	33 (29.5)	5 (20.8)	
Rarely/never	18 (13.2)	14 (12.5)	4 (16.7)	
**Communicates with primary care providers regarding diabetes management**				0.49
Always	11 (8.1)	9 (8.0)	2 (8.3)	
Usually	19 (14.0)	13 (11.6)	6 (25.0)	
Sometimes	58 (42.7)	50 (44.6)	8 (33.3)	
Rarely	32 (23.5)	26 (23.2)	6 (25.0)	
Never	16 (11.8)	14 (12.5)	2 (8.3)	
**Agrees dentist’s role includes diabetes screening**				<0.001
No	27 (19.9)	14 (12.5)	13 (54.2)	
Yes	109 (80.2)	98 (87.5)	11 (45.8)	
**Training received in chairside HbA1c screening**				0.08
None	50 (36.8)	39 (34.8)	11 (45.8)	
Less than one hour	32 (23.5)	28 (25.0)	4 (16.7)	
1-4 hours	29 (21.3)	24 (21.4)	5 (20.8)	
5-8 hours	10 (7.4)	6 (5.4)	4 (16.7)	
More than 8 hours	15 (11.0)	15 (13.4)	0	
**Familiar with billing code for HbA1c screening**				0.60
No	113 (83.1)	93 (83.0)	20 (83.3)	
Yes	23 (16.9)	19 (17.0)	4 (16.7)	
**Agrees monitoring HbA1c can have an impact on diabetes control**				0.003
No	14 (10.3)	7 (6.3)	7 (29.2)	
Yes	122 (89.7)	105 (93.8)	17 (70.8)	

*Numbers may not add up to 100% due to missing data. **Chi-squared and Fisher’s exact tests for bivariate associations.

The overwhelming majority of respondents agreed that dentists’ role includes diabetes screening (80.2%) and that HbA1c monitoring can have an impact on controlling a patient’s diabetes (89.7%). Few were familiar with the billing code for HbA1c screening (16.9%) and 60.3% had zero or less than one hour of training in chairside HbA1c screening.

Overall, 82.4% of respondents were willing to provide HbA1c screening in the dental clinic. Among the providers who agreed that a dentist’s role includes diabetes screening, a greater proportion were willing to screen (87.5%) than among those providers who were not willing to screen (45.8%). Among the providers who were not willing to screen, 29.2% did not feel that HbA1c monitoring had an impact on diabetes control vs 70.8% who did agree that monitoring can have an impact.

Table 2 shows the results of three multivariable logistic regression models for the association of willingness to provide chairside HbA1c screening with three independent variables: (1) “role” (reference = screening is not part of dentist’s role), (2) “impact” (reference = chairside HbA1c monitoring has no impact on diabetes control), and (3) “training” (reference = no training in chairside HbA1c monitoring). Model 1 presents unadjusted models and Model 2 presents fully adjusted models including all independent variables (role, impact, and training) as well as additionally adjusting for age and sex. In both the unadjusted and fully adjusted models, both “role” and “impact” were statistically significantly associated with willingness to provide chairside testing. The odds ratio and 95% confidence interval in the fully adjusted model for “role” were 7.15 and p = 0.001, and for “impact” these were 4.32 and p = 0.04. The independent variable “training” was not statistically significantly associated with willingness to provide chairside HbA1c screening.

**Table 2:** Multivariable logistic regression models of association between exposures of interest (role, impact, and training) and willingness to provide chairside HbA1c screening, N = 136

	Model 1 (unadjusted)		Model 2 (fully adjusted)	
	OR (95% CI)	p-value	OR (95% CI)	p-value
Role (Ref: screening not part of role)	8.27 (3.11, 22.02)	<0.001	7.15 (2.36, 21.63)	0.001
Impact (Ref: monitoring has no impact)	6.18 (1.92, 19.83)	0.002	4.32 (1.07, 17.45)	0.04
Training (Ref: no training)	1.13 (0.76, 1.69)	0.54	1.65 (0.86, 3.17)	0.13

Fully adjusted models include all independent variables (role, impact, and training) and additionally adjusts for age and sex.

In Table 3, the barriers regarding chairside HbA1c monitoring in the dental setting are shown. A majority of respondents agreed or strongly agreed that the amount of time required to obtain and discuss and patient’s test results as well as a lack of reimbursement for the time taken to discuss patient’s results were significant barriers to chairside HbA1c screening (82.3% and 70.6%, respectively). A majority of respondents agreed or strongly agreed that there would be patient resistance to chairside HbA1c testing (35.3% and 18.4% respectively). A majority also agreed or strongly agreed that lack of reimbursement for testing and cost of in-office monitoring equipment and supplies were barriers to chairside testing.

**Table 3:** Barriers regarding chairside HbA1c monitoring in the dental setting, N = 136

Barrier	Strongly agree	Agree	Neither agree nor disagree	Disagree	Strongly disagree
	N (row%)				
1. Amount of time required to obtain and discuss a patient’s test results					
	49 (36.0)	63 (46.3)	13 (9.6)	10 (7.4)	1 (0.7)
2. Lack of reimbursement for the time taken to obtain and discuss a patient’s test result					
	47 (34.6)	49 (36.0)	22 (16.2)	14 (10.3)	4 (2.9)
3. Lack of confidence in my ability to obtain and discuss patient’s test results					
	22 (16.2)	35 (25.7)	21 (15.4)	38 (27.9)	20 (14.7)
4. Patient resistance to having a HbA1c test in the dental office					
	25 (18.4)	48 (35.3)	33 (24.3)	25 (18.4)	5 (3.7)
5. Lack of adequate referral knowledge					
	16 (11.8)	39 (28.7)	25 (18.4)	39 (28.7)	17 (12.5)
6. Lack of insurance reimbursement for testing					
	32 (23.5)	46 (33.8)	35 (25.7)	18 (13.2)	5 (3.7)
7. Cost of in-office monitoring equipment and supplies					
	38 (27.9)	53 (39.0)	26 (19.1)	15 (11.0)	4 (2.9)

## Discussion

We report the results of a study representative of the knowledge and attitudes of dentists working in public health settings in New York City regarding willingness to provide chairside HbA1c screening of patients for diabetes and prediabetes. To our knowledge, this is the first study to look at the attitudes of dentists, both resident and attending dentists, in the largest healthcare system in the United States. The public-health dental setting, where there is often co-location of medical and dental services, offers an untapped and vital venue for intervention to screen for diabetes and reduce the burden of this epidemic.

Our findings, which support previous studies of provider attitudes and experience in private practice and dental schools, suggest that public health dentists understand the value of and are very willing to provide chairside HbA1c screening for dysglycemia.^7,15,21,31,38^ These dentists may, given their practice setting, be more cognizant of the relationship between diabetes and oral disease (i.e. periodontitis, root caries, Candida infection, burning mouth syndrome) as well as the systemic consequences of glucose dysregulation. Our findings corroborate other studies which found that willingness to screen for dysglycemia was strongly associated with the dentists’ belief that it was part of their role as a healthcare professional and that screening would have an impact on diabetes control.^6,47^ However, increased efforts to educate and train all dentists on the impact of oral health on systemic health is needed.^25^ In addition, compensation for dentists’ time in screening and education needs to be addressed in order for providers to be encouraged to incorporate these practices into their workflow.

The finding of a strong association between willingness to screen for diabetes and agreement that screening has an impact on diabetes control is supported by a recent systematic review of clinical and field trials for dysglycemia screening in the dental setting, which concluded that screening effectively identified high-risk patients requiring glycemia management.^2,6,20,23^ Feasibility and pilot studies of chairside A1c screening have found ranges between 34% and 49% of participants with values indicating prediabetes or diabetes, depending on the risk assessment screening tools used for patient selection.^8,9,20,39,48^ Given the high percentage of US adults who see their dentist but not their physician regularly, a recent national study examining National Health and Nutrition Examination Survey (NHANES) data has concluded that screening for prediabetes at dental visits has the potential to alert an estimated 22.4 million adults to their risk of prediabetes or diabetes.^16^

Provider support for HbA1c screening may also stem from endorsements by prominent dental organizations in the US, including the American Dental Association and American Dental Education Association. These organizations advocate screening of multiple chronic diseases, including diabetes, cardiovascular disease, hypertension, and human immunodeficiency virus infection (HIV).^22,25,29,30^ The American Dental Association supports the point of care prediabetes identification guide co-developed by the Centers for Disease Control and Prevention, American Medical Association, and American Diabetes Association (available at https://www.cdc.gov/diabetes/prevention/pdf/prediabetes-screening-test-tag508.pdf).

An additional consideration with regard to point-of-care testing for dysglycemia is the cost-effectiveness of screening in a dental setting. A recent study in the US used simulation modelling to evaluate the cost-effectiveness of point-of-care screening in a dental setting for dysglycemic patients being managed by a weight reduction intervention.^35^ Based on the standard of approximately $50,000-60,000 per quality-adjusted life year (QALY),^18^ they concluded that screening was not only cost-effective but also cost-saving compared to other approaches. A study from the Netherlands supports the use of a step-wise screening approach for prediabetes and diabetes in which only those high-risk individuals who exceed a pre-determined threshold (based on a questionnaire) are tested in order to prevent wasteful spending of resources.^12^

Consistent with previous literature, the most commonly perceived barriers to willingness to perform chairside HbA1c testing were cost/reimbursement and time.^3,24,32,35,46^ These barriers may explain why, in our study, even among those providers who agreed that monitoring HbA1c can have an impact on diabetes control, that 71% were still not willing to perform the testing. A possible remedy for the issue of time may be increased and novel use of auxiliary dental professionals such as dental hygienists, dental therapists, and community dental health coordinators in the chairside screening workflow. In addition, it is critical that an operational, and preferably electronic, bidirectional referral pathway exists between dental clinics and primary medical care departments so that medical diagnosis, management, and follow-up can occur.

The strengths of our study include a high survey response rate (52%) that offered insight into the knowledge and attitudes of public health dentists working with populations at high risk for diabetes in New York City. Limitations of our study are the lack of generalizability to dentists working in private practice settings and/or with patient populations of higher socioeconomic levels. In addition, our findings may not be generalizable to dentists outside of New York State (due to potential differences in state practice laws) nor to dentists outside of the US, where the healthcare systems operate differently with regard to access to care and cost factors.

## Conclusion

Our findings suggest that there is overall high acceptance of the use of chairside HbA1c testing in the public health dental setting and that dental providers’ willingness to perform the testing is strongly associated with their belief that it is part of their role as a healthcare provider. However, in order to see increased utilization of chairside testing, our study suggests that it is not additional training that will necessarily help, but that processes must be addressed so that the barriers of the providers’ time and associated costs are minimized. Future research to address implementation strategies utilizing auxiliary dental providers in public health settings is encouraged in order to gain broader acceptance for chairside diabetes screening and facilitate greater implementation by dentists both in public health settings and private practice.
